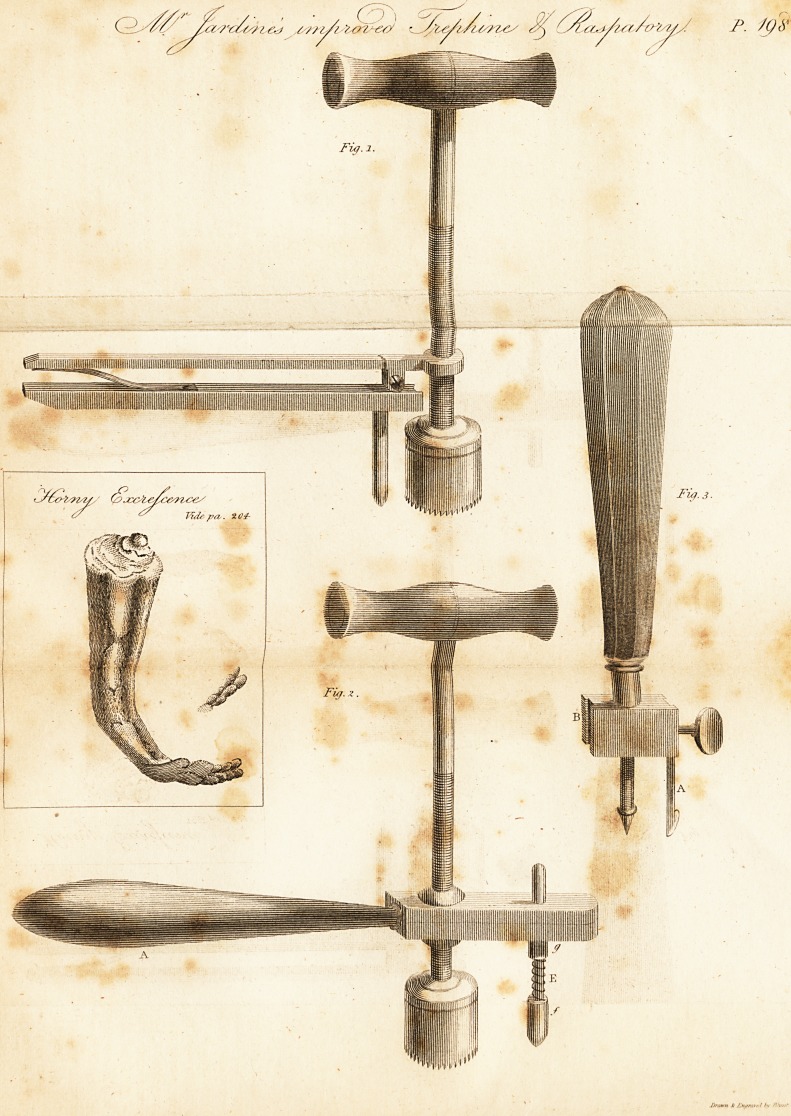# Mr. Jardine, on Surgical Instruments

**Published:** 1804-09-01

**Authors:** W. Jardine

**Affiliations:** Dumfries


### *To the Editors of the Medical and Phyſical Journal.*

GENTLEMEN,

IN the course of my practice as a Surgeon in the Navy, I could not help observing the very defective form of many Surgical instruments used in the most important operations. Whatever therefore occurred to me as improvements in that way, I left a manuscript account of with Mr. Savigny, Surgeon's Instrument Maker, in London, which he undertook to get inserted in the Medical Journal there, as was accordingly done, in that periodical work for December 1802.

Since

Fig. 1.

Fig. 3.

Horny Excrescence

Vide pa. 204

Fig. 2.

Drawn & Engraved by Blunt

Since that time it has frequently been observed to me, that the complicateness of the form of some of the improvements alluded to, not only makes them very expensive to the purchaser, but troublesome in the application. I have endeavoured, for this reason, to simplify those, which from such considerations may be thought the most exceptionable.

In the form I now offer them, they may not only be made much cheaper but more manageable, and, of course, more generally useful.

If what I have done will but afford a useful hint to others more capable of such improvements, I shall be satisfied; being rather apprehensive, that from my present state of health, I shall not be able, by future experience in surgery, to prosecute such improvements further.

I wish to avail myself of this opportunity, likewise, in correcting a few of the principal alterations and substitutions, that have been made without my knowledge in the aforesaid manuscript: such corrections being, in my opinion, absolutely necessary in some cases, to explain what I really had in view, as I am rather doubtful that the description you received will be scarcely, if at all, understood by the reader.

Presuming therefore that you will allow me that satisfaction, I begin, following the order in the Journal, with some prefatory observations I made on the management of Instruments in general.

1st. In No. 46, p. 482, for the twenty inferior lines of the second paragraph, instead of "to obviate, &c."—read as follows:

The observation, I confess, is too true, but may, I think, be easily accounted for. It is a rule with artificers to accustom their apprentices to the use of their tools, before they allow them to do any thing but what is conducive thereto; in that way they acquire a certain dexterity in the handling them, which they would not otherwise have; and even others, which they may have occasion at a future period to make use of, although they should be new and unknown to them before.

Were young surgeons equally industrious in accustoming themselves, in a similar way, to the use of their instruments, upon such different substances as their instruments are intended to operate upon, in place of dead bodies, which but a few can have an opportunity of, they would not be so much at a loss in managing any thing new as they often are.

2. In place of "*The noblest of all professions,*" an epithet particularly introduced in the alterations made.—If any epithet is thought necessary to advance the rank or merit of the profession, I have no objection to dignify it with the appellation of being one of *the most useful and necessary.*

3. Page 483, paragraph iii.—For, the thread of the screw upon the legs of the trevet should be coarse, in order to regulate its position or necessary variations *with more celerity;* read, the screw-thread upon the feet, to fit the trevet to its place *the sooner,* should be as coarse as possible.

4. Page 484, paragraph iii.—For, may answer the purpose of a levator, &c.—read, may answer the purpose of a levator with another shank, having a coarser screw upon it, and a suitable piece with a rotatory motion on the end of it, which, in the operation, should be placed under the depression; the handle of the instrument, in that case, being turned the opposite way from what it should be in making the perforation.

5. Page 486, paragraph iv. and line 5.—If he can there "introduce the instrument"—read, If he can there *thrust* in the instrument, or if the very decayed state of the tooth or stump does not forbid the application of it to that particular place.

6. Page 487, paragraph i.—last five lines wholly superadded.

7. Paragraph iii. line 1.—For *contracted,* read *extended;* line 6,—for extended, read spread.

8. Page 487. The following notice is given, immediately after the description of the instrument for extracting bones, pins, &c. in the œsophagus, viz. "The above instrument, with Mr. Cruikshank's admirable contrivance for extracting pieces of money, &c. and the sponge probang, arranged in a compact case, are at all times to be met with at Savigny's."

From the above notification, it may be inferred that the instrument I have recommended, will not answer the same purpose as Mr. Cruikshanks's.

I would be glad to know what objection there can be against using of my instrument in extracting a piece of money, as well as any other substance that should not be forced down into the stomach. It will surely require less dexterity in spreading the cat-gut under a piece of money to to be extracted, than in catching it with a hook; and in withdrawing the instrument, it will more readily be brought up, therefore it must answer all the ends proposed by Mr. Cruikshanks, and consequently supersede its use.

The more readily to understand the meaning of the improvements now proposed, it is necessary to refer the reader, who is not acquainted with my former designs, to your Journal for December 1802.

In place of the trevet or triangular rest for suspending the saw, and preventing it, in the operation of the trepan, from slipping suddenly down upon the brain, a kind of machinery that must unavoidably require some time in adjusting it to the unequal surface of the head; the danger dreaded in that operation may as certainly be avoided, in a much more simple way, by a rectangular prop, one side of which is to form the foot and the other the handle. The foot to be pointed at the lower end, and to rest perpendicularly upon the cranium. The lever should be of the same length as the handle, and move upon it by a pivot near the angle.

It is not necessary that the screw upon the shanks of the trephine should be so very fine as in the former design; a coarser one, of about seventy or eighty threads to the inch, I dare say, will answer the end proposed very well.

The fineness of the screw upon the shanks, not only makes it very liable to be broken, but considerably enhances the price of the instrument.

In working the trephine upon the latter plan, the operator having previously made a small hole near the place to be perforated, to receive the pointed end of the foot, takes a firm hold, with his left hand, of the handle of the support and lever; and with his right hand, the handle of the trephine; the saw, in the course of the operation, is raised or depressed at any particular point, merely by the inclination of the prop and motion of the lever, through which the shank of the trephine is screwed.

The preference of the latter design to the former, must be very obvious; from the latter instrument being at once fitted to the place to be operated upon, requiring less time in its necessary variations during the operation, and by resting more firmly upon the bone than the former can upon the teguments, it will be wrought more steadily.

It has occurred to me, since writing the above, that the trephine may likewise be very aptly prevented from slipping suddenly down upon the brain, in the operation of the trepan, merely by its shank, either plain or with a screw screw upon it, passing through a ball confined in a socket in the upper or horizontal portion of such a rectangular support as is now recommended in preference of the trevet, with a spring foot to it to rest upon the cranium, and wrought in the above manner.

The last method of guarding the trephine is not only very simple, but, I think, will be found the most manageable of any I have yet thought of.

No surgeon of experience, in wounds and contusions of the head, I presume, will object to the instrument resting upon the bone in this operation as is here proposed, knowing, that any material injury done the head, is occasioned by the violence or force with which the blow is given.—However, any surgeon disliking that method of steadying the instrument, may very easily fix a cushion to the foot of it, which he may rest either on the bone or teguments, as he thinks proper.

*The Raspatory.*

Two trepanning saws of different sizes being all the variety required in general practice, the raspatory may be made to answer its end more readily in a much more simple and cheaper manner, than according to the design in your Journal for December, 1802.

In place of the adjusting slide, the blades may be made to slide on both sides of the shank, proportioning the distance between the centre-pin and blade, on one side, to the diameter of the largest saw; and the distance between the centre-pin and blade on the other side, to the diameter of the smallest saw, and the blade in use to be secured by a screw for that purpose,

Care should be taken in forming the blades of the raspatory, that they may not be made broader than necessary; for the fore and back part describing two different circles, in perforating a part of the circle, which, in some cases, the operator may wish to do, the back part of the blade will rub so much against the side of the perforation as to be checked in its progress.

Surgeons, who do not think proper to make use of the raspatory, according to its original intention, will find this instrument very suitable for removing any small portion of the cranium, or of any other bone, or where a narrow or small opening is required which cannot be made by the trephine. This instrument, by placing the centre-pin in two different places, will make such an opening as may be intended.

If

If this instrument can be so usefully employed, in place of one so very unfit for its purpose, it cannot well be considered as making many unnecessary additions to the instruments for the operation in question.

In any attempt to make the trepanning instruments as portable as possible, and to diminish their number, the different blades of the raspatory may be made to answer the purpose of that instrument by making them to slide perpendicularly in the circumference of the saw, and to project occasionally beyond the teeth, as far as the operator, for any of the particular purposes mentioned, may think proper.

*Lenticular.*

I have been informed, that some very respectable surgeons now seldom, if ever, make use of the lenticular, completing the perforation at once with the saw.

How it is possible to complete the perforation with the saw, of a bone in some parts so very unequal in its thickness as the cranium, and furrowed so deeply in some places within by the blood-vessels of the dura mater, without cutting some of them, or otherwise materially injuring that membrane, which we know likewise adheres very fast to the internal surface of the bone, I cannot conceive —Were the operator certain of the membrane being detached, by a collection of matter betwixt it and the scull, he might consider such an instrument, in completing the perforation unnecessary; but until some rule is laid down, by which we can be assured of the membrane being separated from the bone, the perforation cannot be completed by the saw, but at the risk of injuring the dura mater, or cutting some of its vessels.

*Dumfries,* I am, &c.
*April* 12, 1804. W. JARDINE.

---

EXPLANATION OF THE PLATE.

Fig. 1. A view of the trephine suspended by a rectangular prop instead of a trevet.

Fig. 2. A side view of the shanks of the trephine screwed through the ball in a socket.

E. the foot with a spring surrounding a spindle that passes easily through the top.—One end of the spring is fixed at *f.* and the other to the ring *g.*

Fig. 3. The raspatory—A. the blade fixed in the slide to form the largest circle— B. the slide for receiving the blade, to form the smallest circle.

P. S. Mr. Savigny, surely, could not have misunderstood me so much, when I gave him permission to publish the description description of the instruments I left with him, as to think that he had permission from me to alter the manuscript as he thought proper. No, that was not my design; but as he was in doubt at first whether it would be best to publish an account of them in a pamphlet, or in the Medical Journal of London; I allowed him, in any manner he thought proper, that is, either in the Medical Journal of London, or in a pamphlet by itself.

## Figures and Tables

**Fig. 1. Fig. 2. Fig. 3. f1:**